# Reply to ‘Experimental measurement of assembly indices are required to determine the threshold for life’

**DOI:** 10.1098/rsif.2024.0622

**Published:** 2024-11-20

**Authors:** Robert M. Hazen, Peter C. Burns, H. James Cleaves II, Robert T. Downs, Sergey V. Krivovichev, Michael L. Wong

**Affiliations:** ^1^ Earth and Planets Laboratory, Carnegie Institution for Science, Washington, DC 20015, USA; ^2^ Department of Civil and Environmental Engineering and Earth Sciences, Department of Chemistry and Biochemistry, University of Notre Dame, Notre Dame, IN 46556, USA; ^3^ Earth Life Science Institute, Tokyo Institute of Technology, Tokyo 152-8550, Japan; ^4^ Blue Marble Space Institute for Science, Seattle, WA 98104, USA; ^5^ Department of Chemistry, Howard University, Washington, DC 20059, USA; ^6^ Geological Sciences, University of Arizona, Tucson, AZ 85721, USA; ^7^ Department of Crystallography, Institute of Earth Sciences, St Petersburg State University, St Petersburg 199034, Russia; ^8^ Nanomaterials Research Centre, Kola Science Centre, Russian Academy of Sciences, Fersmma 14, Apatity 184209, Russia; ^9^ Sagan Fellow, NASA Hubble Fellowship Program, Space Telescope Science Institute, Baltimore, MD 21218, USA

**Keywords:** assembly theory, assembly indices, evolution, mineral complexity, heteropolyanion, mineral evolution

## Abstract

We clarify misunderstandings of Walker et al. (Walker *et al.* 2024 *J. R. Soc. Interface* 21, 20240367 (doi:10.1098/rsif.2024.0367)) related to studies of the assembly pathways of molecular subunits in minerals. The finding that these subunits have calculated assembly pathways less than approximately 25 informs a central premise of Assembly Theory—that only life can produce numerous copies of molecules with assembly indices above a threshold value. What that threshold value might be, and whether the same value applies to chemical systems as different as organic and inorganic molecules, are questions deserving of additional study.

*J. R. Soc. Interface* **21**: 20240367 (Published online 20 November 2024). (https://doi.org/10.1098/rsif.2024.0367)

Cronin, Walker, and colleagues *et al*. [1–8] have championed Assembly Theory (AT), which posits that two attributes serve to distinguish complex products of living systems from non-living objects: (i) living systems produce objects requiring numerous assembly steps and (ii) the objects are present in numerous copies. According to this hypothesis, only a directed assembly by life can overcome the combinatorial improbability of such complex objects.

In their comment [9], Walker *et al*. suggest that our recent work [10] misunderstands and/or misrepresents aspects of AT. While the originators of AT are in the best position to recognize mischaracterizations of their theory, and we are grateful for any corrections or clarifications of their publications, we wish to correct several of their own misunderstandings of our contribution.

1. Contrary to the claims of Walker *et al*. [9], we did not consider mineral lattices, which we agree are not amenable to AT analysis because they are continuously connected, space-filling periodic structures. Rather, we focused exclusively on discrete molecules—heteropolyanions that occur abundantly as isolated units in solution and that under appropriate evaporative conditions may become incorporated into crystal lattices [11–13]. These objects, as required for AT evaluation, are both present in large copy numbers and are ‘finite, distinguishable, persist in time, and [are] breakable’. We conclude that heteropolyanions are amenable to precise assembly pathway determination.
2. Walker *et al*. [9] explain that the assembly index (AI) is the ‘shortest number of recursive steps to construct an object’, whereas assembly pathway is ‘the shortest pathway to construct the object’. Their assertion is that AIs must be determined experimentally, whereas assembly pathways are calculable. This point seems to contradict statements in their own publications that one ‘can calculate the assembly index’ [3], that offer ‘algorithms we have developed to calculate [molecular assembly]’ [7], and that one can use ‘assembly index calculations on polymeric strings’ [6]. Nevertheless, with tutoring from two authors of the ‘Comment’ (i.e., e-mails from S. I. Walker; Zoom and e-mail discussions with C. Marshall), we employed the ‘Molecular assembly [Python 3 calculator]’ [7] and associated instructions and illustrated examples [1–6] to calculate minimum assembly pathways (not AIs) of several heteropolyanions. By definition, since the assembly pathway is the shortest possible construction pathway, the procedures we employed [1–7] allowed us to define the minimum possible AI for each mineral subunit. We conclude that the actual AIs of some heteropolyanions in natural systems, which may be greater but not less than the calculated minima of assembly pathway values, are greater than 20 for some inorganic abiotic molecules that occur in large copy numbers. In this regard, we acknowledge that inorganic mineral heteropolyanions represent a different class of molecules from the organic molecules studied by Cronin, Walker, and colleagues; therefore, a different maximum value for abiotic systems is plausible.
3. There is no compelling evidence that the heteropolyanions we considered ‘are very unstable’ [9]. On the contrary, these and similar heteropolyanions are sufficiently stable that they can be generated in large numbers and studied in solution [11–14]. Furthermore, minerals such as ewingite, paddlewheelite and ilmajokite, which incorporate these complex molecule-like sub-assemblies, form in crystals up to 1 mm diameter from evaporating solutions (https://rruff.info/ima and references therein; accessed 3 September 2024), suggesting the presence of at least 10^9^ molecular copies in localized natural solutions. However, we agree that further studies of these intriguing solutions and molecular copy numbers are warranted.
4. Walker *et al*. [9] argue that the chemical bonding of heteropolyanions and organic molecules is fundamentally different, because only organic molecules feature covalent bonds. This assertion echoes an often- misunderstood aspect of bonding in minerals. While it is true that the bonding in oxygen-based minerals is often (for convenience) couched in terms of cations and anions, experimental electron density studies and theoretical calculations demonstrate that the chemical bonding in these molecule-like units is significantly covalent in nature [15,16]. In particular, the U–O, V–O, C–O, and/or Si–O bonds featured in all mineral examples we analysed [10] are predominantly covalent in character. We based our assembly pathway calculations on these covalent bonds (not individual atoms), employing the same methods as Cronin, Walker, *et al*. in their publications [1–7].
5. Walker *et al*. [9] correctly note that minerals such as ewingite, paddlewheelite, and ilmajokite are described in our previous work as ‘biologically mediated’—a reference to the critical role of oxygenic photosynthesis in creating a near-surface oxidizing environment on Earth [17]. However, as we have emphasized [10], microbes played no role in the nucleation and growth of these heteropolyanions or the crystals in which they are incorporated. Any planet or moon with similarly oxidizing chemical environments would have the potential to produce these complex mineral phases.

If AT is to be developed as a valid and useful concept, and we agree that its core ideas are both plausible and in principle verifiable, then it should apply to the assembly of any kind of molecular unit in nature, including the complex heteropolyanions that are sometimes incorporated in large copy numbers into minerals. If, on the other hand, AT only applies to organic molecules, then it cannot serve as the sole and unique basis for an agnostic biosignature.

Therefore, we stress again that our work on mineral complexity supports a core conclusion of AT: while some mineral heteropolyanions have minimum assembly indices significantly greater than the value of 15 cited by Walker and colleagues *et al*. [2], there appears to be a limit to the AIs of these abiotic molecules of less than 25—a limit greatly exceeded by some biomolecules. Furthermore, recent work has demonstrated that minerals represent a bounded evolving system [18], possibly in contrast to life based on organic chemistry as an open-ended evolving system [19–21]. In this context, AT offers the prospect of quantifying the molecular manifestation of life’s open-ended combinatorial richness.

## Data Availability

This article has no additional data.
